# A Common Psychology of Male Violence? Assessing the Effects of Misogyny on Intentions to Engage in Violent Extremism, Interpersonal Violence and Support for Violence against Women

**DOI:** 10.1080/09546553.2023.2292723

**Published:** 2024-01-02

**Authors:** Bettina Rottweiler, Caitlin Clemmow, Paul Gill

**Affiliations:** Security and Crime Science Department, University College London, London, UK

**Keywords:** Misogyny, violent extremism, terrorism, violence against women and girls, gender-based violence, hypermasculinity, collective narcissism, revenge motivation

## Abstract

The growing evidence base of risk factors for violent extremism demonstrates overlaps with different types of gender-based violent behaviours, such as intimate partner violence, sexual assault, and sexual harassment. Each of these manifestations of violence are, to a varying extent, underpinned by misogynistic and hypermasculine attitudes and behaviours. The present analysis aims to address the limited empirical research on the links between misogyny, violent extremism, and gender-based violence by conducting survey-based analyses employing a newly developed and validated psychometric scale to measure misogyny. Based on a U.K. nationally representative survey (*n* = 1500), we examine the underlying mechanisms and contingent effects linking misogyny to violent extremism, interpersonal violence, and violence against women. The results show that misogyny predicts violent extremist intentions, willingness to engage in interpersonal violence and increased support for violence against women via revenge planning and hypermasculinity, particularly among men who experience a sense of violated entitlement and greater threats to the ingroup. Among women, misogyny is not associated with violent extremist intentions but is associated with readiness to use interpersonal violence and with increased support for violence against women. Our findings largely suggest a common psychology underlying different types of male violence. This has important practical implications, (1) suggesting that misogyny is a shared risk factor which underpins different types of male violence, (2) highlighting the mechanisms which link misogyny to (extremist) violence, (3) while further providing evidence which articulates when and for whom misogyny may be a risk factor. Establishing the relevance of misogyny as a risk factor for extremist and gender-based violence provides evidence pointing towards the potential benefits of incorporating misogyny within existing (extremist) risk assessment tools. Identifying shared mechanisms via which misogyny exerts its effects on different forms of male-perpetrated violence, further offers initial evidence to inform programmatic approaches to prevent and counter gender-based and targeted violence.

## Introduction

“There can be no doubt—patriarchy, misogyny, domestic abuse and mass murder are associated, and have been for a long time. That these links were identified not by criminologists but by activists and female journalists suggests that popular criminology is surging ahead of academic research and the latter needs to catch up.”^1^

Public discourse on misogyny and its harmful consequences—not just for women and girls but also for men and wider society—is growing. Broad-based social movements (e.g., #MeToo), violence prevention awareness programs, and highly publicised instances of sexual harassment and sexual assault increasingly bring discussions of misogyny and related constructs (e.g., hostile sexism, toxic masculinity) to the fore. This increased media and policy interest has further been fuelled by newly emerging threats (i.e., Involuntary Celibates “incels” and the “manosphere”)^2^ as well as recent high-profile attacks which were either motivated by a grievance toward women and/ or the perpetrator had a known history of violence against women and girls (VAWG).^3^

Established empirical research demonstrates that misogynistic attitudes and extreme adherence to patriarchal beliefs underpin different manifestations of gender-based violence in the domestic sphere, such as intimate partner violence, stalking, sexual harassment, and sexual assault.^4^ Increasingly, we are also witnessing a shift in academic and policy discourse towards recognising that grievances and resentment against women often present fundamental motives underpinning various acts of “public” violence, including terrorism and other acts of targeted violence.^5^ Importantly, critical terrorism studies and feminist scholars have long demanded for policy and practice to adopt a “gender lens” to address violent extremism^6^ and to better understand how male violence in the public and domestic spheres are likely intertwined.^7^

Pain^8^ notes both, domestic violence (also referred to as “everyday terrorism,” “intimate terrorism,” and “patriarchal terrorism”) and violent extremism, try to exert power, dominance and control through fear and violence.^9^ Both are rooted in patriarchal gender roles, which justify the policing and punishment of women who violate men’s gendered expectations.^10^ Relatedly, a growing body of work in neighbouring research domains suggests that previous engagement in “private” violence, including VAWG and intimate partner violence, presents a pertinent risk factor for engagement in targeted violence. For instance, McCulloch et al. challenge the dichotomy in how we understand and how we respond to violence against women and more public-facing forms of violence, such as acts of targeted violence and terrorism.^11^ Accordingly, terrorism “blurs the boundaries between public and private, known and unknown perpetrators, collective and individual motivations, and so on, and is mediated by masculinity.”^12^

Emerging empirical research further highlights misogynistic attitudes and behaviours are outwardly expressed in diverse forms of extremism such as far-right extremism, jihadism and among “misogynistic incels.”^13^ Male supremacist and strict patriarchal norms and gender roles are common elements which underpin different extremist ideologies, strategies, and tactics, whereby misogyny is used to justify violence against women in order to reinforce male-dominated gendered hierarchies.^14^ Correspondingly, a growing body of work finds grievances against women and a history of gender-based violence are common among violent extremists and terrorists.^15^ Similarly, research consistently shows that perpetrators of other forms^16^ of targeted violence are often motivated by grievances against women and/ or share histories of gender-based violence, such as domestic violence,^17^ sexual assault,^18^ stalking,^19^ and online harassment of women.^20^

Collectively, this suggests that different forms of targeted violence are likely, to some extent, intertwined and intersect with gender-based violence in the domestic sphere, whereby they appear to share a common psychology which is underpinned by grievances towards women.^21^ These grievances are commonly rooted in misogynistic attitudes and patriarchal norms, driven by a sense of injustice due to thwarted entitlement over women who deny men their desired “dominant” status in the gender hierarchy and thus, violate patriarchal gender roles. Resultingly, engagement in revengeful and hypermasculine behaviours are common ways to redress such grievances. Based on this emerging empirical evidence, domestic and intimate partner violence have been identified as warning signs for potential acts of targeted violence.^22^

## Context

“Most misogynistic behavior is about hostility toward women who violate patriarchal norms and expectations, who aren’t serving male interests in the ways they’re expected to.”^23^

A culture of misogyny often forms a core part of different types of male violence, whether it is public violence or private violence. Violent crimes against women fuelled by misogyny have been most evident among several recent acts of targeted violence in the U.S., U.K., and Canada. Such attacks highlight the *functional* role misogyny can play in motivating acts of mass violence. Some of these perpetrators identified, to varying degrees, as “incels” and/ or engaged in misogynistic online communities prior to committing the attacks.^24^ Incels are a loosely organised online community of men who are unable to attract women sexually. Incels typically consider themselves to be at the bottom of society, and while they still feel entitled to women’s bodies, they perceive to be denied access to sexual and romantic relationships.^25^ As a result, they commonly express hostile attitudes and blame women, and more specifically feminism, as well as attractive and sexually active men for their sexual and romantic rejection and celibacy.^26^

While not all incels hold violent attitudes towards women and seek vengeance, “misogynistic incels” regularly call for violence against women.^27^ A minority of these individuals have gone on to commit large-scale acts of violence, whereby misogyny has been enacted by indiscriminately targeting individuals who are symbolic of a whole group in society, that is women.^28^ Gentry^29^ describes incel violence as a form of terrorism grounded in an ideology based upon misogynistic ideals and values, whereby the aim is the subjugation and repression of women to (re)impose male-dominated hierarchies.^30^ By externalising their anger and hatred, stemming from a deep sense of violated entitlement, misogynistic incels engage in violent masculinities through acts of mass violence.^31^

The case of Elliot Rodger, one of the most well-known misogynistic mass murderers and posthumously labelled as an incel, particularly highlights how romantic and sexual rejection can induce a sense of violated entitlement and a perceived injustice among men.^32^ His own perceived superiority transformed shame and anger into a desire for revenge against women, but also against sexually active men, ethnic minorities, and wider society.^33^ The manifesto and videos produced by Rodger were a fundamental part in the rise of the misogynist incel movement shaped around sexual entitlement, the dehumanisation of women and the glorification and perpetration of male violence. Resultingly, this has inspired several incel-related misogynistic attacks within recent years,^34^ prompting the National Threat Assessment Center/United States Secret Service and the U.S. Department of Homeland Security^35^ to label these “newly emerging” threats as “misogynistic extremism.”

Although attention in regard to the threat stemming from misogynistic belief systems often predominantly focuses on the incel movement, misogyny, threats to masculinity and a sense of violated male entitlement go far beyond the incel community and present common grievances which underpin different types of male private and public violence. For instance, Gentry’s critical analysis on terrorism research claims “misogynistic terrorism” has always been there, while further arguing that the vast majority of the research community is still reluctant to acknowledge the underpinning element of misogyny in regard to far-right extremism.^36^ She further criticises the “[…] inability or resistance to see misogyny and its related violence that targets women as a political ideology”^37^ and calls for such violent acts to be treated as terrorism.^38^ As such, the term “misogynistic terrorism” encompasses a body of scholarship which argues misogyny and its related forms of violence against women are in fact political and driven by an ideology, labelling it violent extremism in its own right that seeks to police and enforce women’s subordination to uphold a patriarchal political system. Thus, the subjugation and repression of women is similar to that of other groups in society, whereby personal and/or group-based grievances drive different manifestations of male violence. More specifically, in the case of misogynistic extremism, this grievance is explicitly related to hostile attitudes and a resentment towards women, but nevertheless, is often intermingled with other grievances.^39^

Yet, violent extremism and terrorism research, with its focus on *political* and *ideological* violence, is hesitant to acknowledge that misogyny and its violent manifestations, e.g., gender-based violence, are inherently ideological as well as political. Feminist scholars argue violent misogyny presents a way of punishing and policing women to uphold patriarchal norms and rigid traditional gender roles and thus, to enforce male dominance and female submission.^40^ Thus, in its very essence, misogyny involves *ideological* and *political* elements, as it supports patriarchal structures and norms in society which shape political and social institutions, rules, laws as well as social and cultural norms and values.^41^

Relatedly, recent research argues misogynistic belief systems form a core part of different violent extremist ideologies, such as jihadism and far-right extremism.^42^ Misogyny is rooted in extreme patriarchal norms, including male dominance/ male supremacism and female (sexual) subjugation, which have been identified as fundamentally shared values across different extremist ideologies, i.e., far-right and jihadist extremism as well as male supremacism and “inceldom.”^43^ Roose and Cook examined actors across the ideological spectrum and note a shared “desire to impose extreme patriarchal social and political order with male supremacism as a primary goal, where violence against women, and the institutions seen to be advancing gender equality, are justified, and are inherent in their ideologies.”^44^ Recent survey research across Asia and North Africa finds that support for violence against women, followed by hostile sexist attitudes, are the strongest predictors of support for violent extremism. Individuals who demonstrated support for VAWG were three times more likely to endorse violent extremism.^45^

Similarly, qualitative studies highlight histories of domestic abuse and documented misogyny in the backgrounds of terrorists within Western countries.^46^ Windisch’s findings on sixty-eight lone actor terrorists from 2001–2016 suggest that prior to committing terrorism, many perpetrators engaged in forms of gender-based violence to assert a dominant form of masculinity.^47^ This is in line with research, which argues that extreme misogyny, grievances against women, e.g., a sense of violated entitlement toward (white) women and sex, and a desire for women’s subjugation have been fundamental to perpetrators’ motivation.^48^ Relatedly, blaming feminism for the falling birth rates in the West, perceived existential threats to the white race, i.e., white genocide, and a sense of perceived decline of white male hegemony have been common antecedents amongst far-right terrorists, which ultimately have led to a desire to engage in retributive violence.^49^

A grievance against women has further been prevalent among other forms of targeted violence, which have not been classified as violent extremism or terrorism, nor was the perpetrator affiliated or inspired by the incel movement, but nevertheless, either espoused a strong grievance against women and/ or had committed gender-based violence prior to engaging in mass violence. Regarding the former, Silva et al.’s finding reveal that 34 percent of all public mass shootings in the U.S. between 1966 and 2018 (*n* = 106) and 45 percent (*n* = 48) between 2010 and 2018 were motivated by grievances against women, whereby the perpetrators were engaging in taking “corrective action” in the form of violence.^50^ Such statistics highlight the importance of considering the role of gender more broadly and misogyny as well as “crisis of masculinity/ toxic masculinity” more specifically in explaining mass shootings,^51^ as “[…] perhaps the most extreme version of performative violent masculinity.”^52^

Regarding the latter, recent research demonstrates that intimate partner and/ or domestic violence oftentimes present precipitating factors for mass shootings.^53^ For instance, Marganski’s study on mass murder events in the U.S. finds that 83 percent of men engaged in some form of violence against women prior to committing mass murder.^54^ Very similar findings emerge from DeVoe and Nicholson’s review of mass shootings in the U.S. spanning the period between 1966 and 2020, which shows that nearly half of all public mass shooters had a history of previous engagement in VAWG.^55^ Similarly, Geller et al.^56^ show that between 2014 and 2019, 68 percent of mass shooting offenders either killed at least one partner or family member and/or had known histories of previous engagement in domestic violence, while other studies find that 53 percent of mass shootings involved the perpetrator killing a family member or intimate partner, in addition to other victims.^57^

Importantly, these incidents point towards significant overlaps in the motivational underpinnings of different forms of male violence, whether it is violence against women in the domestic sphere or acts of targeted, public-facing violence. They highlight the role of misogyny, violated entitlement beliefs, masculinity threats and a desire for revenge as fundamental motivations driving various acts of violence.^58^ Such attitudes and grievances as well as motivations to “do gender” and to engage in “corrective” action, have been studied for decades by gender scholars in relation to gender-based “private” violence. A wealth of research has linked the need for male dominance, power, and control to a range of adverse outcomes, i.e., intimate partner violence/ domestic violence, coercive behaviours, and rape proclivity among men, yet rarely are these concepts applied to studying acts of targeted “public” violence, such as those committed by mass murderers, misogynistic incels and violent extremists.^59^

Despite recent developments, policy makers as well as mainstream discourse still tend to shy away from applying a *gendered approach* to explaining and debating acts of targeted violence, including terrorism.^60^ This may be in part due to limited systematic research that explores whether there are in fact overlaps and commonalities linking violent extremism and other types of public violence with private violence among men, and it still remains unclear whether misogyny is indeed part of a common psychology driving male violence. Furthermore, there is even less empirical research which examines the complex motivational processes and underlying mechanisms, which may be able to specify *when* and *how* misogyny may be relevant to violent extremist risk and violence against women and thus, may underpin different manifestations of male violence.

To explore the above, quantitative studies which provide robust empirical evidence are essential, yet are extremely limited to date. Quantitative investigations are necessary because they help identify relative effect sizes against other competing contributory factors, model how other factors mediate, exacerbate, or dampen these effects, identify which sub-sets of the population are more prone to experience these effects, provide risk assessment with empirically evidenced risk factors to inform judgements about the level of risk posed, and help in the formulation of most suitable primary and secondary intervention strands. This is not to suggest quantitative accounts hold more value than qualitative ones but rather they help shine a light on different parts of the problem. Whilst the qualitative accounts to date provide us with rich case-specific knowledge on the functional relevance of misogyny in the expression of violent extremism and other forms of targeted violence, robustly designed quantitative accounts help us understand the presence, imminence, and salience of the relationship at a broader level and the potential degree of generalisability that qualitative accounts hold.

In part, the current quantitative gap in knowledge may also be due to difficulties in measuring and operationalising misogyny for quantitative research. Hence, the present study employs a newly developed and validated psychometric scale to examine the relationship between misogyny and the outcomes (1) intentions to engage in violent extremist behaviours, (2) willingness to engage in interpersonal violence, and (3) support for violence against women. We test several moderated mediation models to explore how and when/ for whom misogyny predicts the different violent outcomes. More specifically, we examine whether the effects of misogyny on all three outcome measures are mediated through (1) revenge motivation and (2) hypermasculinity among men. We further test whether the mediations through revenge and through hypermasculinity are contingent upon men’s levels of collective narcissism (violated entitlement) and men’s threat perceptions. This allows us to specify *how* and *when* misogyny may be relevant to violent extremism, interpersonal violence and violence against women and thus, may underpin different manifestations of male violence.

## Theoretical rationale

In the coming sub-sections, we present our theoretical rationale. In particular, we focus upon the potential roles of collective narcissism, revenge motivation, hypermasculinity and threat perceptions in promoting support for different types of male violence.

### Collective narcissism

Collective narcissism concerns violated group-based entitlement and is defined as “a belief that one’s own group (the ingroup) is exceptional and entitled to special recognition and privileged treatment, but it is not sufficiently recognized by others.”^61^ Research on collective narcissism offers ways to understand how frustrated group entitlement beliefs and perceived group injustice can induce outgroup hostility and revenge thoughts. In turn, this may lead to violence perpetration.

More specifically, collective narcissists are emotionally invested in a grandiose image of the ingroup, which requires constant validation. This idealised image is unstable and thus vulnerable to internal and external threats.^62^ Resultingly, collective narcissists tend to view the actions of others as threatening, which in turn renders them prone to aggressively engage in intergroup hostility to protect and defend the ingroup’s status.^63^ Indiscriminately targeting any out-group member thus appears to be one way to restore threatened in-group and self-image amongst collective narcissists.^64^ Importantly, male collective narcissism is associated with viewing women as a threatening out-group resulting in both, less empathy and greater hostile sexism towards women.^65^ In the context of ethnic conflict, collective narcissism has been shown to predict support for violent extremism as measured by (1) support for violence to attain a separate Tamil state, (2) support for ideological violence in service of Islam, and (3) support for armed Jihad.^66^

### Revengefulness

“Revengefulness” is defined as the tendency to uphold and to engage in revenge thoughts, which may translate into retributive violence as well as the inability to forgive prior offences.^67^ A desire for revenge has been described as a fundamental motive in terrorism,^68^ homicide,^69^ and rape,^70^ alike. Studies demonstrate the links between revengefulness, high levels of entitlement and collective narcissism.^71^ Others demonstrate fantasies of vengeful murder, which are linked to entitled frustration and blame externalisation,^72^ while studies show that individuals with stronger revenge thoughts are indeed more likely to harm others.^73^ In a similar vein, Cooper et al.’s findings indicate overlaps regarding the motivational underpinnings of fatal family violence and acts of targeted violence, whereby both were found to be predominantly motivated by grievance and a desire for revenge.^74^ This aligns with research suggesting that revenge offers wronged individuals a sense of empowerment in which justice and the moral balance can be restored,^75^ and whereby dominance and masculinity can be reinforced.^76^

### Hypermasculinity

The above aligns with research on masculinity, group status, and threats. These areas provide valuable explanations of how threats to masculine identity may transform into hypermasculine behaviours and extreme outgroup derogation, and thus, may contribute to mass and gender-based violence.^77^ Hypermasculinity refers to an extreme adherence to stereotypical masculine gender roles. Hypermasculinity is operationalised with three main characteristics: a belief that violence is manly, callous sex attitudes, and the view that danger is exciting.^78^ Hypermasculinity often results in endorsing positions that subjugate and devalue women, engaging in violent fantasises about, and/or conducting aggressive behaviours toward women.^79^ For a variety of reasons, these effects are particularly strong when men perceive their gender status to be threatened or challenged.^80^

First, aggression helps to down-regulate negative emotions which can arise when their manhood is challenged.^81^ Second, by engaging in hypermasculine behaviours and by derogating and subjugating women, men reinforce their power and dominant status within the patriarchal gender hierarchy as well as against outgroups.^82^ This suggests men who perceive their masculine gender identity to be threatened may, in some cases, turn to aggression, violence, and supremacist beliefs to perform dominant and violent masculinity.^83^ Relatedly, Scaptura et al.’s study on far-right violent extremist offenders in the U.S. highlights ways in which homicides were rooted in strained dominant masculinities, whereby extremist violence and violence against women commonly intersected.^84^ Scaptura et al. conclude that shared underlying social-psychological mechanisms, such as power and control, may in fact shape two seemingly distinct forms of violence in a similar way.^85^

### Hypotheses

**Figure 1.**
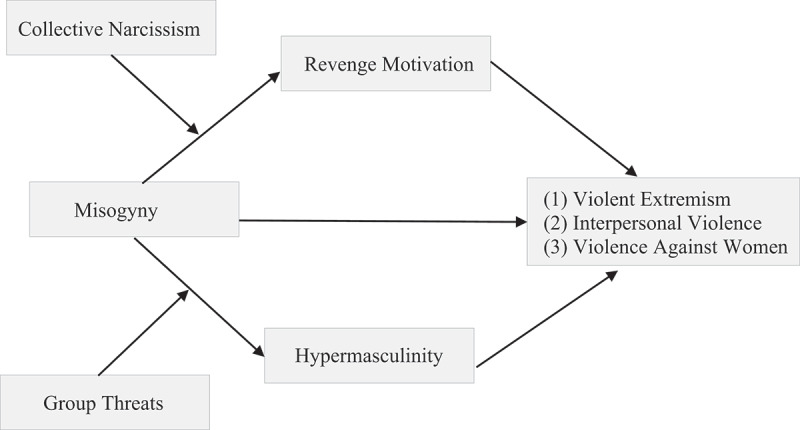
Path diagram to present the hypothesised moderated mediations among men with: (1) Misogyny as independent variable, revenge motivation as mediator, the a-path moderated by collective narcissism and violent extremist intentions, willingness to engage in interpersonal violence, and support for violence against women as outcome criteria. (2) Misogyny as independent variable, hypermasculinity as mediator, the a-path moderated by group threat and violent extremist intentions, willingness to engage in interpersonal violence, and support for violence against women as outcome criteria.

The above accounts suggest that misogyny is associated with male violence, i.e., violent extremism, interpersonal violence, and violence against women. We expect this relationship to be mediated by revenge motivation particularly among men who hold high levels of collective narcissism and as such feel that men as a group are not sufficiently recognised despite the group’s perceived exceptionality, resulting in a sense of violated narcissistic entitlement, which requires retributive violence (Figure 1).^86^ Therefore, it is hypothesised that:

**H1**: Men who hold misogynistic beliefs show an increased *willingness to engage in violent extremism*, whereby the effects are mediated via heightened revenge-seeking behaviour. The indirect (i.e., mediated) effects are particularly strong for those men who hold high levels of collective narcissism (i.e., violated entitlement beliefs).

**H2**: Men who hold misogynistic beliefs demonstrate an increased *willingness to engage in interpersonal violence*, whereby the effects are mediated via heightened revenge-seeking behaviour. The indirect effects are particularly strong for those men who hold high levels of collective narcissism.

**H3**: Men who hold misogynistic beliefs show stronger *support for violence against women*, whereby the effects are mediated via heightened revenge-seeking behaviour. The indirect effects are particularly strong for those men who hold high levels of collective narcissism.

It is further expected that the relationships between misogyny and the violent outcome measures are mediated via hypermasculinity, particularly among men who perceive their ingroup to be threatened. As mentioned above, threats to the ingroup may motivate hyper-conformity to stereotypical masculine traits to demonstrate dominant masculinity and to “do gender,” which in turn, increase the risk towards violent behaviours (Figure 1).^87^ Therefore, it is hypothesised that:

**H4**: Men who hold misogynistic beliefs show an increased *willingness to engage in violent extremism*, whereby the effects are mediated via hypermasculine attitudes. The indirect effects are particularly strong for those men who hold high levels of perceived threats to their ingroup.

**H5**: Men who hold misogynistic beliefs demonstrate an increased *willingness to engage in interpersonal violence*, whereby the effects are mediated via hypermasculine attitudes. The indirect effects are particularly strong for those men who hold high levels of perceived threats to their ingroup.

**H6**: Men who hold misogynistic beliefs show stronger *support for violence against women*, whereby the effects are mediated via hypermasculine attitudes. The indirect effects are particularly strong for those men who hold high levels of perceived threats to their ingroup.

## Method

### Participants

Data collection took place in July 2020. Participants were recruited via the online platform Prolific. Participants were based on a U.K. nationally representative sample (by age, gender, and ethnicity) *n* = 1500. Overall, 51.3 percent (*n* = 769) identified as female, 48.7 percent (*n* = 730) identified as male, and one individual indicated non-binary as their gender status (*M*_ageM_ = 44.52; *SD*_ageM_ = 16.21; *M*_ageW_ = 45.31; *SD*_ageW_ = 15.62). See Table 1 for a breakdown of ethnicity and education among female and male participants.

**Table 1.** Sociodemographic characteristics of participants

		Men	Women
Characteristics		*n* (%)	*n* (%)
Ethnicity			
	Asian	57 (7.8 percent)	58 (7.5 percent)
	Black	25 (3.4 percent)	30 (3.9 percent)
	Mixed	15 (2.1 percent)	16 (2.1 percent)
	Other	13 (1.8 percent)	11 (1.4 percent)
	White	618 (84.9 percent)	654 (85 percent)
	Total	730	769
Education			
	No formal qualifications	(1.9 percent)	(2.1 percent)
	GCSEs	(18.6 percent)	(17 percent)
	A-levels/BTEC	(27.4 percent)	(23.7 percent)
	Undergraduate Degree	(35.8 percent)	(40.1 percent)
	Masters	(13 percent)	(14.6 percent)
	Completed PhD	(3.2 percent)	(2.6 percent)
	Total	730	769

### Procedure

Participants were invited to participate in a study on risk and protective factors for violent extremism. After completing the consent form, participants were asked to fill out the questionnaire. After data collection finished, the data were examined to ensure data quality. We assessed whether respondents had missed attention checks as well as the completion time for each participant. Participants were excluded from the data analysis if they missed more than two attention checks and if they completed the survey more than two standard deviations quicker than the average completion time.

### Statistical analyses

To test the hypotheses, we examined path models with moderated mediations in Lavaan.^88^ Age and education were entered as covariates (the analyses without the covariates yielded almost the same results). The models were run with 5000 bootstrap samples and 95 percent bias corrected bootstrap confidence intervals as this method is robust to non-parametric data and statistical outliers. Further, this method effectively handles deviations from the normal distribution of study variables as no assumptions about the shape of the sampling distribution are made.^89^ Bootstrapping is a non-parametric technique and there is growing consensus that bootstrapping is the preferred resampling strategy for estimation and hypothesis testing, particularly when testing for indirect and conditional indirect effects.^90^

To examine the hypotheses that the effects of the relationship between misogyny and the violent outcome variables are (1) mediated by revenge planning and are conditional upon levels of collective narcissism and are (2) mediated by hypermasculinity and are contingent on perceived group threats among men, two separate moderated mediation path models were estimated. We ran the first moderated mediation model for the revenge mediator and the collective narcissism moderator including all three outcome variables in one model. The second model was run for the hypermasculinity mediator and the group threats moderator including all three outcome variables. For both models, the path (a-path) linking misogyny to (1) revenge planning is expected to be moderated by collective narcissism, and the path (a-path) linking misogyny to (2) hypermasculinity is expected to be moderated by group threats, indicating that the effects are strongest for those highest in collective narcissism and group threats, respectively, which subsequently renders the overall indirect effects dependent on levels of the moderators. Similar to analyses conducted with PROCESS, inferential tests for the moderation of the indirect effects were estimated (index of moderated mediation).^91^ The moderated mediation index estimates how differences in misogyny result in differences in the outcome variables indirectly through revenge planning and hypermasculinity and depending on the values of collective narcissism and group threats. If the indirect effect differs systematically as a function of the moderator, i.e., the confidence interval of the index does not contain zero, then the mediation is said to be moderated.^92^

### Measures

Unless stated otherwise, all items were measured on seven-point Likert scales ranging from 1 (strongly disagree) to 7 (strongly agree). For all scales, the individual items were averaged to calculate a score for each individual, whereby higher values denoted, for example, stronger misogynistic attitudes, higher levels of collective narcissism, and stronger intentions to engage in violent extremism etc.

#### Misogyny

In this paper, we use a measure which defines misogyny as the hostility and distrust towards or devaluation of women or girls. To measure misogyny, we employed a newly developed ten-item psychometric scale (e.g., “Women exploit men for their own agendas,” “When it comes down to it a lot of women are deceitful” ω_Men_ = .95, ω_Women_ = .93, see preprint here: https://psyarxiv.com/6f829).^93^ The three-factor misogyny scale (i.e., “Manipulative and exploitative nature of women,” “Distrust towards women,” “Devaluation of women”) was developed and validated across three studies based on a U.K. nationally representative survey. Construct and measurement validity were established across several studies. An exploratory factor analysis established the factor structure of the ten-item misogyny scale. Subsequently, the ten-item structure was replicated via confirmatory factor analysis. The misogyny scale displayed good convergent (i.e., significant and strong relationship with male sexual entitlement, masculinity related violent beliefs and willingness to use violence) and high internal consistency (composite reliability). Additionally, measurement invariance across gender and age groups was established, allowing researchers to deploy the scale among male and female individuals, across different age groups and to assess latent mean differences.

#### Revenge motivation

Revenge motivation was operationalised with the validated “Revenge Planning” subscale of the “Displaced Aggression Questionnaire,” which measures the tendency to hold a grudge for a prior provocation and a plan for retaliation and thoughts and ideas of retribution (e.g., “When someone makes me angry, I can’t stop thinking about how to get back at this person,” ω_Men_ = .95, ω_Women_ = .95).^94^ The scale showed excellent internal consistency and test–retest reliability and demonstrated convergent as well as discriminant construct validity.

#### Hypermasculinity

Hypermasculinity was measured with four items from the “Machismo” subscale of the “Maudsley Violence Questionnaire,” which is a validated measure that has demonstrated construct validity across several studies (e.g., “Sometimes you have to be violent to show that you are a man,” ω_Men_ = .82, ω_Women_ = .76).^95^ “Machismo” items describe violence as manly, similar to Mosher and Sirkin’s Hypermasculinity Inventory.^96^ The items refer to stereotypical expectations of men pertaining to toughness and manliness. Such attitudes indicate that aggression and violence are not only expected but are desirable as they are means to express strength and assertiveness among men.

#### Collective narcissism

We operationalised the five-item Collective Narcissism Scale (e.g., “I will never be satisfied until my group gets the recognition it deserves,” ω_Men_ = .84, ω_Women_ = .89).^97^ The collective narcissism scale has demonstrated strong internal reliability across several studies^98^ as well as convergent, divergent, and predictive validity.^99^

#### Group threats

Perceived group threats among men were measured with a single item “I have the feeling that the group I belong to is under threat in the U.K.,” which was developed for this study. We employed three different outcome variables to measure support and willingness to engage in different types of violence among men.

#### Violent extremist intentions

Violent extremist intentions was assessed with the four-item Radical Intention Scale (RIS), which is a validated and widely used scale to measure individuals’ willingness to engage in illegal and violent actions on behalf of a group (e.g., “I would continue to support an organization that fights for my group’s political and legal rights even if the organization sometimes resorts to violence,” “I would participate in a public protest against the oppression of my group even if I thought the protest might turn violent,” ω_Men_ =.84, ω_Women_ = .81).^100^ The scale showed good internal validity and confirmatory factor analysis established construct validity.^101^

#### Willingness to use violence

Items were part of a larger violence scale development and were inspired by the “Acceptance of Violence” subscale of the “Maudsley Violence Questionnaire.”^102^ The interpersonal violence measure captured participants willingness to use or engage in violence and was assessed with three items (e.g., “If someone provokes me, I’ll respond with my fists,” “In certain situations, I’m willing to use violence,” ω_Men_ = .76; ω_Women_ = .75).

#### Support for violence against women

Endorsement of violence against women was measured with three items, tapping into participants’ support for violent behaviours towards women and intimate partners (e.g., “It is ok for a man to use violence against a woman if she misbehaves,” “It is ok for men to use force against their partner in order to keep authority in their relationship,” ω_Men_ = .85, ω_Women_ = .71). We ran confirmatory factor analyses (CFA) on all scale measures. All CFAs showed satisfactory fit indices and all indicators showed adequate factor loadings with standardised coefficients ranging from *β* = .58 to *β* = .95.

## Results

Table 2 shows the separate correlations among all variables for men and women. As expected, the correlations between misogyny and the other study variables were all stronger for men compared to women, apart from the correlation between misogyny and support for violence against women, which was unexpectedly stronger for women. Amongst men, misogyny demonstrates a positive and significant association of small to moderate strength with all variables. All other operationalised variables are positively and significantly correlated with each other, indicating medium to strong effect sizes among men. Further, we examined whether misogyny is associated with the violent extremism, support for VAWG and interpersonal violence measures amongst our female sample. The correlation results show that misogyny is *not* correlated with violent extremist intentions (*r* = .00, *p* > .05), among women. Yet, for women misogyny demonstrates a significant positive relationship with intentions to engage in interpersonal violence and importantly, equally with support for violence against women. Interestingly, we find stronger correlations between support for violence against women and (1) misogyny, (2) hypermasculinity, (3) collective narcissism, and (4) willingness to engage in interpersonal violence for the female sample. The remaining variables for the female sample are all significant and show small to medium effect sizes with one another. Mean difference tests between genders revealed that men demonstrate significantly higher means than women for all variables operationalised, ranging from small to large effect sizes (also see Table 2).

**H1**: **–3—Moderated Mediation 1:** Misogyny, Revenge Planning, Collective Narcissism, on (1) Violent Extremist Intentions, (2) Willingness to Engage in Interpersonal Violence, and (3) Support for Violence against Women, among Men

**Table 2.** Descriptive statistics and correlations among men and women

Variables	Men	Women	Mean differences			Correlations							
	*M* (*SD*)	*M* (*SD*)	*T*	*p*	*d*	1	2	3	4	5	6	7	8
1. Misogyny	2.63 (1.17)	2.23 (1.06)	6.45	<.001	.33	–	.37***	.36***	.15***	.16***	.00	.26***	.28***
2. Revenge motivation	2.69 (1.34)	2.37 (1.30)	4.79	<.001	.25	.48***	–	.42***	.36***	.18***	.23***	.46***	.21***
3. Hypermasculinity	1.97 (1.00)	1.50 (.68)	10.71	<.001	.55	.42***	.49***	–	.26***	.17***	.27***	.67***	.33***
4. Collective narcissism	3.31 (1.23)	3.20 (1.20)	2.12	<.05	.11	.34***	.45***	.31***	–	.38***	.23***	.24***	.14***
5. Group threat	2.94 (1.78)	2.75 (1.63)	2.12	<.05	.10	.43***	.34***	.34***	.42***	–	.25***	.20***	.11**
6. Violent extremist intentions	2.82 (1.36)	2.54 (1.21)	4.23	<.001	.22	.17***	.34***	.38***	.25***	.22***	–	.37***	.11**
7. Willingness to use violence	2.19 (1.13)	1.53 (.82)	13.08	<.001	.68	.43***	.55***	.69***	.33***	.30***	.44***	–	.41***
8. Support for violence against women	1.11 (.45)	1.05 (.24)	3.34	<.001	.17	.23***	.24***	.27***	.10**	.20***	.14***	.33***	–

Pearson’s correlation coefficients are reported. Correlations for women are presented above, and correlations for men below the diagonal of the correlation matrix.

*n* = 1499 (*n*_women _= 769; *n*_men _= 730).

*p < .05. **p < .01. ***p < .001.

We examined if and how misogyny predicts (1) violent extremist intentions, (2) willingness to engage in interpersonal violence, and (3) support for violence against women directly and indirectly through the mediator revenge planning. We further examined whether the indirect effects are contingent upon men’s levels of collective narcissism. The a-path linking misogyny to revenge planning is expected to be moderated by levels of collective narcissism. Overall, our first hypothesised model has a very good fit: CFI = .989, TLI = .968, RMSEA = .043, and SRMR = .022. Misogyny (*a*_1_ = .31, 95 percent CI [.16, .48]) and collective narcissism (*a*_2_ = .13, 95 percent CI [.01, .25]) are significant predictors of revenge motivation and their interaction is also significant (*a*_3_ = .04, 95 percent CI [.01, .08]). Revenge motivation, in turn, shows a significant and positive effect on violent extremist intentions (*b*_1_ = .34, 95 percent CI [.26, .42]), willingness to engage in interpersonal violence (*b*_2_ = .40, 95 percent CI [.34, .46]), and support for violence against women (*b*_3_ = .06, 95 percent CI [.03, .08]).

Additionally, we examined the conditional indirect effects at the three levels of the moderator (i.e., at the mean as well as M−/+ 1SD). Table 3 shows the indices of the moderated mediations. The results demonstrate that, as hypothesised, the effects of misogyny on violent extremist intentions, willingness to engage in interpersonal violence, and VAWG are mediated via revenge planning and are strongest for those men who experience higher levels of collective narcissism, indicated by a significant moderated mediation effect (supporting **H1**—**H3**). The direct effect of misogyny on violent extremist intentions is not significant when the mediator and control variables are estimated simultaneously (*c*’_1_ = .01, 95 percent CI [−.08, .10]). The direct effect of misogyny on intentions to engage in interpersonal violence (*c*’_2_ = .20, 95 percent CI [.13, .25]) and VAWG (*c*’_3_ = .05, 95 percent CI [.02, .08]) are statically significant after including revenge motivation and covariates in the model.

**Table 3.** Misogyny predicting violent (extremist) outcome measures among men

Mediator		Criterion	Index of moderated mediation	Conditional indirect effects at level of (1) collective narcissism (2) group threats			Hypothesis Supported
				Low	Moderate	High	Yes/ No
Collective Narcissism				(1)			
**H1**		Violent extremist intentions	.01 [.001, .02]	.13 [.08, .18]	.15 [.10, .20]	.17 [.12, .23]	Yes
**H2**		Willingness to engage in interpersonal violence	.02 [.01, .03]	.15 [.10, .21]	.17 [.13, .22]	.20 [.15, .25]	Yes
**H3**		Support for violence against women	.004 [.001, .008]	.02 [.01, .04]	.02 [.01, .04]	.03 [.02, .05]	Yes
Perceived Threats				(2)			
**H4**		Violent extremist intentions	.02 [.01, .03]	.12 [.09, .16]	.15 [.10, .20]	.18 [.13, .23]	Yes
**H5**		Willingness to engage in interpersonal violence	.02 [.003, .04]	.16 [.10, .23]	.20 [.15, .26]	.24 [.18, .31]	Yes
**H6**		Support for violence against women	.01 [.001, .20]	.03 [.02, .05]	.04 [.02, .06]	.05 [.03, .07]	Yes

Misogyny predicting violent (extremist) outcome measures among men, mediated by revenge motivation or hypermasculinity, whereby the a-paths are moderated by collective narcissism or perceived group threats. Total, direct, and indirect effects of misogyny predicting violent outcomes among men, mediated by revenge planning or perceived group threats. 95 percent bias-corrected confidence intervals used, along with 5000 bootstrap samples. Controlling for age and education. Index of moderated mediation = statistical test for the moderation of the indirect effects. Levels of the moderators are M–1SD (low levels of collective narcissism/ group threats), M (average levels of collective narcissism/ group threats), and M + 1SD (high levels collective narcissism/group threats).

These findings indicate that misogyny predicts (1) violent extremist intentions, (2) willingness to engage in interpersonal violence, and (3) support for VAWG indirectly via increased revenge thoughts and the effects are particularly strong for men who hold high levels of violated entitlement beliefs.

**H4: –6—Moderated Mediation 2:** Misogyny, Hypermasculinity, Group Threat, on (1) Violent Extremist Intentions, (2) Willingness to Engage in Interpersonal Violence, and (3) Support for Violence against Women, among Men

We further tested if and how misogyny predicts (1) violent extremist intentions, (2) willingness to engage in interpersonal violence, and (3) support for violence against women, directly and indirectly through the mediator hypermasculinity. We further tested whether the indirect effects are dependent on levels of perceived group threats. The a-path from misogyny to hypermasculinity is expected to be moderated by levels of threat perceptions. Our second hypothesised model has a good fit: CFI = .987, TLI = .960, RMSEA = .053, and SRMR = .028.

The results show that misogyny shows a significant positive effect on hypermasculinity (*a*_1_ = .20, 95 percent CI [.19, .31]), whereas the main effects of perceived group threats are non-significant (*a*_2_ = .02, 95 percent CI [−.07, .10]). However, the interaction between misogyny and perceived threats on hypermasculinity is statistically significant (*a*_3_ = .03, 95 percent CI [.01, .06]). Hypermasculinity, in turn, is a significant predictor for violent extremist intentions (*b*_1_ = .52, 95 percent CI [.42, .62]), willingness to engage in interpersonal violence (*b*_2_ = .71, 95 percent CI [.64, .77]), and VAWG (*b*_3_ = .13, 95 percent CI [.10, .16]).

The indices for the moderated mediations are statistically significant, indicating that the effects of misogyny on all three violent outcome measures are mediated via hypermasculinity and are moderated by levels of group threats (supporting **H4–H6**). The findings show that the indirect effects are strongest for those men who report higher levels of group threats. While the direct effect of misogyny on violent extremism is not significant (*c*’_1_ = −.01, 95 percent CI [−.10, .07]), the direct effects of misogyny on willingness to engage in interpersonal violence (*c*’_2_ = .14, 95 percent CI [.09, .20]) and VAWG (*c*’_3_ = .04, 95 percent CI [.01, .06]) are statistically significant after the effects of revenge thoughts and covariates are controlled for.

## Discussion

Previous qualitative studies provide initial evidence which suggests that different forms of targeted violence, including acts of mass violence and violent extremism/ terrorism, are to some extent intertwined with gender-based violence,^103^ while separate work has started to examine the role of misogyny within violent extremist ideologies^104^ and among incels.^105^ Despite this important qualitative body of research, there has been a dearth of empirical work examining the potential interconnectedness underlying violence against women and violent extremism and thus, exploring whether these manifestations present *distinct* or *related* forms of male violence. Therefore, we examined whether similar mechanisms underpin men’s motivations to commit different types of violence, i.e., violent extremism, VAWG and general interpersonal violence and most importantly, whether these are rooted in misogyny. Overall, our findings demonstrate that misogyny plays a functional role in underpinning different forms of violence among men, shedding light on *how* and *when* grievances against women can potentially motivate violent behaviours among men.

Going beyond simply establishing misogyny as a predictor for different types of male violence, we articulate the *relevance* of misogyny by examining the mediating and moderating effects linking misogyny, violent extremism, violence against women and interpersonal violence. Interestingly, the present study shows that both men and women endorse misogynistic attitudes, but the consequences differ. While misogyny was significantly and positively associated with violent extremist intentions among men, this relationship was not significant among women. Yet, misogyny was associated with increased support for violence against women and readiness to use violence, amongst women. Importantly, we find stronger correlations between support for VAWG and (1) misogyny, (2) hypermasculinity, (3) collective narcissism, and (4) willingness to engage in interpersonal violence in the female sample compared to the male sample. This suggests that hostile attitudes towards women and factors tapping into strict gendered attributes, such as hypermasculinity, potentially play a more functional role in justifying violence against women among women themselves. However, this hypothesis requires further testing.

Overall, our findings provide tentative support that men’s support and willingness to engage in different forms of violent behaviours in both, the private sphere (i.e., interpersonal violence and violence against women), and the public sphere (i.e., violent extremism), are underpinned by similar risk factors and mechanisms. These findings stand in contrast to what we find among women. While providing initial evidence, further empirical testing and scrutiny is required to test for differential risk factors and pathways among genders.

We further examined how and when misogyny, group threats, and violated narcissistic entitlement translate into revenge-seeking behaviour and hypermasculinity, and thus, increase readiness to engage in extremist and interpersonal violence as well as support for violence against women, among men. Our findings suggest that violent extremism, interpersonal violence, and violence against women may be partly driven by misogyny, revenge motivation, and hypermasculinity. More specifically, the first conditional mediation model showed that men with stronger misogynistic beliefs and higher levels of violated entitlement are more likely to engage in revenge planning, which in turn, predicts a stronger willingness to engage in violent extremism and interpersonal violence as well as stronger support for violence against women.

More generally, engaging in misogyny may help to uplift men’s self/group image and worth, particularly if women are perceived to threaten traditional gender norms and hierarchies.^106^ Hence, the use of violence may be linked to men’s resentment toward their perceived loss of dominance and masculinity, in which revenge is seen to restore power and control. Our results are in line with studies that found hostile sexism among men to be related to (male) collective narcissism,^107^ while collective narcissism was further associated with retaliatory intergroup aggression^108^ and an increased desire for revenge, particularly against threatening outgroups.^109^ Relatedly, recent survey studies finds that collective narcissism is positively associated with support for violent extremist ideologies and groups.^110^ Our findings further align with qualitative research on violent extremism suggesting that perceived threats to men’s status within the gender hierarchy can lead to feelings of injustice, violated entitlement and a sense of victimhood.^111^ The resultant resentment toward women has been shown to turn men’s anger into a desire for revenge against women, ethnic minorities but also society as a whole.^112^ According to Roose, “Men who subscribe to this ideology believe that women’s empowerment has left them victimized and discriminated against. They play out their anger and resentment through violent acts, justifying these as merely reclaiming what they believe is rightfully theirs.”^113^

The second conditional mediation analysis showed that when misogynistic men perceive their ingroup to be threatened, they exhibit stronger hypermasculine attitudes (e.g., justifying violence and emphasising male dominance and strength), which in turn, predicts higher levels of support for violence against women and stronger intentions to engage in violent extremism as well as interpersonal violence. These findings are consistent with the idea that adopting a violent masculinity and executing violent retribution may represent means of demonstrating or restoring dominant masculinity, particularly if men experience group or status threats.^114^ Extreme misogyny, grounded in patriarchal norms and systems, appears to drive, to some extent, these violent expressions of masculinities. The study findings also align with previous research which argues for a link between misogyny, masculinity, and violence within extremist groups^115^ and work highlighting the patriarchal nature of jihadist and far-right ideologies that is tied to a strict adherence to masculine gender and cultural ideals.^116^ Correspondingly, engagement in violent extremist groups has been argued to demonstrate a way of exhibiting male dominance, whereby the use of violence is a means to assert a dominant masculine status.^117^ Attempts to reinforce male dominance have further been evident among more recent phenomena, such as on- and offline sexual harassment and trolling campaigns, whereby men perform “masculinity” with one another through hostile, hateful and derogatory behaviours towards women.^118^

Two key implications of our findings are worth highlighting. First, our results show that revenge motivation and hypermasculinity present two crucial mechanisms demonstrating *how* misogynistic attitudes may translate into a vulnerability to engage in different types of male violence. Second, our findings further suggest that to understand this complex relationship, in addition to understanding how misogyny drives revenge motivation and hypermasculinity, it is key to examine *when* or *for whom* this is particularly relevant. Therefore, such evidence of how and when/ for whom a risk factor, here misogyny, is functionally linked to an adverse outcome via the mechanisms at work, is crucial for identifying potential prevention and intervention strategies against different types of male violence.

Collectively, our findings specify how misogyny relates to risk towards engagement in different types of violence, suggesting that there are similar psychological factors and mechanisms underlying seemingly different forms of vulnerability to engagement in male violence, calling into question whether they are in fact that distinct. We term these shared characteristics as being part of a so-called “common psychology.” This common psychology seems to underlie the formation of a grievance specifically revolving around the resentment of women, whereby misogyny provides the “ideological” justification, motivating and driving extreme forms of performative violence not just against women but also against wider society. Such a shared psychology sheds light on the potential mechanisms which may explain why violent extremists often have histories of interpersonal violence and in particular, intimate partner violence. Overall, this supports research conducted by Hamby & Grych^119^ showing that different forms of violence often co-occur because they share common risk factors, indicating similar underlying mechanisms at play that drive different types of (male) violence.^120^

Importantly, the relationship between misogyny, violence against women and violent extremism will not be fully understood until there is a more holistic and at the same time more nuanced understanding of the range of psychological mechanisms that underpin these types of behaviours, establishing where they are similar and where they are distinct. This suggests that while it is vital to identify shared risk factors, it is equally important to establish what might differentiate “private” violence against women from public-facing, targeted violence. While private and public acts of male violence seem to be related, they are certainly not co-extensive. Thus, future studies should explore which psychological mechanisms underlie the separate occurrence and co-occurrence of violence against women and violent extremism among males.^121^ Additionally, rather than assessing those grievances and strains in isolation, it is imperative to analyse the complex constellation, interplay, and dynamicity of factors to get closer to understanding when and why, what combination of risk and protective factors are relevant. Importantly, while the co-occurrence of shared risk factors and mechanisms among these previously assumed distinct types of violence does suggest a common psychology, for the vast majority of individuals these risk factors being present will not have any relevance upon violent outcomes due to the multifinality of risk factors.^122^

### Practical implications

Collectively, our findings indicate that misogyny is not only, as previously established, a risk factor for violence against women, but we demonstrate how misogyny underpins and is at the same time blurring the lines between various forms of male perpetrated private and public violence.^123^ This has important practical implications. For instance, this suggests that violence against women can equally present a risk factor for violent extremism and vice versa due to their shared risk markers and underlying causes, rendering men who are vulnerable to violent extremism also more vulnerable to engagement in different types of gender-based violence. This aligns with research conducted by the Violence Prevention Network and the Centre for Feminist Foreign Policy that suggests gender-based violence presents a warning sign for ideologically motivated acts of violence.^124^ Importantly, we argue for a non-causal relationship, meaning that while these types of extremist and non-extremist violence share common risk factors and causes, for the majority of cases engagement in intimate partner violence and other types of violence against women do *not cause* violent extremism. Instead, engagement in violence against women increases one’s *vulnerability* to exposure to and engagement in violent extremism and vice versa.

Therefore, establishing a robust evidence base of the risk and protective factors for misogyny and violent masculinity is key for preventing male violence and equally for understanding susceptibility to extremist groups, ideologies, and narratives. For instance, men who engage in gendered violence are more prone to endorse and internalise misogynistic and hypermasculine attitudes. This in turn, increases their susceptibility and exposure to extremist narratives and settings and thus, their risk towards engagement in violent extremism, rendering misogyny and VAWG pertinent risk indicators for violent extremism. Therefore, understanding what makes boys and men “vulnerable” to misogyny will help to address patterns of underlying risk factors driving different types of harmful behaviours and outcomes, that are not just harmful for women but equally for men themselves. Resultingly, our findings suggest that there is a rationale for more research on the overlaps and common psychology driving male violence, while also highlighting opportunities for practitioners to integrate these empirical findings into the prevention and management of different forms of male violence. Particularly, the interconnectedness of different types of male violence provides opportunities for better safeguarding as well as shared prevention and intervention efforts. Relatedly, there is growing consensus that the relationship between misogyny, acts of gendered violence and violent extremism is worth further exploration and there is tentative evidence of the benefits of incorporating these concepts within existing P/CVE programs.^125^

Considering prevention, combatting violent extremism and targeted violence continues to evolve towards a public health approach, aiming to mitigate vulnerability before behaviour escalates. For instance, the PREVENT arm of the U.K.’s counterterrorism strategy (CONTEST),^126^ as well as the Department of Homeland Security’s (DHS) recently announced Center for Prevention Programs and Partnerships (CP3),^127^ employ a public health approach to combatting grievance-fuelled violence. In regard to “early” prevention and related preventative public health approaches, protective factors are key to inform the design and delivery of primary prevention and early interventions. Interventions focussing on developing and strengthening protective factors may be particularly beneficial to prevent the onset of risk factors as well as to exert buffering protective effects against pertinent risk factors, which may dampen, mitigate, or nullify potential risk.

Therefore, early preventative programming implemented within educational settings, sports clubs, and other recreational settings, may benefit from incorporating interventions which target a variety of general protective factors that can elicit protective functions against a range of different types of (male) violence as well as other adverse behaviours. In addition, given the relationship between misogyny and violent extremism, as well as gendered violence more generally, preventative programming aimed at challenging misogynistic and violent masculine attitudes may be of substantial benefit in terms of violence and crime prevention. The implications are likely to be far wider-reaching than grievance-fuelled violence only, given the gendered nature of crime and violence in general, including sexual assault, intimate partner violence, and stalking.

For instance, programmes incorporating positive role models and aiming to build respectful, healthy, and inclusive forms of masculinity within male-dominated spaces have shown positive effects. Other efforts involving transformative gender strategies,^128^ which ultimately seek to transform gender inequalities as well as challenge stereotypical masculine norms and rigid gender roles, may be particularly relevant for young people.^129^ Thus, comprehensive gender mainstreaming efforts are vital to address gendered dynamics and gender power relations of female subjugation and male dominance.^130^ The most effective programmes for sustained gender transformation have shown to involve both males and females in order to change patriarchal gender norms and promote more gender-equitable relations between men and women.^131^ In addition, fostering tolerance, empathy, and mutual understanding, while at the same time addressing mental issues and personal insecurities, may help to develop healthy confidence and stable self-esteem, creating more resilient young people. Collectively, these approaches may exert positive and protective effects across a range of harms. For instance, a recent study on self-reported domestic abuse perpetration and violent extremist intentions in the U.K. shows internal locus of control, resilience, and self-esteem can exert protective effects upon both types of violence, demonstrating the potential for shared prevention efforts.^132^

Considering secondary prevention, intervention programs targeting vulnerable individuals or those “at-risk” whose grievance involves misogyny or support for violence against women, may benefit most from trying to lessen potential risk by strengthening interactive protective factors, which aim to buffer risk effects. Potential interventions may be based upon developing positive social support systems, such as working with mentors or involvement in voluntary community efforts.^133^ Additionally, fostering forgiveness and emotional regulation has been shown to down-regulate negative affect and anger rumination, which in turn, has been associated with increased capacity to execute self-control and thus, may be beneficial to prevent violent behaviour.^134^ In a similar vein, recent research on different violent extremist outcomes highlights trait forgiveness, a strong capacity for self-control as well as critical and open-minded thinking styles can lessen violent extremist risk among those individuals who experience a sense group-based violated entitlement and injustice.^135^ Collectively, this indicates that (1) direct protective factors can lessen overall risk towards different types of violence and (2) interactive protective effects can dampen violent (extremist) risk, and thus may be highly relevant for some people, especially those “at-risk.”^136^

Risk assessment and in particular, Structured Professional judgment (SPJ) tools, require information-gathering and rely on a formulation-based approach to inform judgements about the level of risk posed, ideally informed by empirically evidenced risk and protective factors and the mechanisms/ pathways through which they exert their effects and interact to drive risk. Existing violent extremist risk assessment tools, such as the Vulnerability Assessment Framework (VAF)^137^ used across PREVENT in the U.K., the VERA-2 R^138^ and the ERG 22+ implemented in prisons to assess risk of violent extremist recidivism, do not explicitly consider misogyny, masculinity or gendered dynamics so far.^139^ Yet, our findings provide initial empirical evidence to support incorporating misogyny and gender dimensions into existing SPJ guidance.^140^ Given our findings, practitioners may wish to consider misogyny relevant to the risk assessment of potential violent extremist offenders, particularly when expressed alongside hypermasculinity and revenge-seeking behaviours and amongst those who express violated entitlement and perceived threats. The present results could equally inform SPJ guidance developed to assess and manage the risk of domestic and gendered violence. For example, it broadens the types of (distorted/negative/violent) attitudes requiring attention for the Short-Term Assessment of Risk and Treatability,^141^ SAVRY,^142^ SARA-v3,^143^ and HCR-20v3^144^ guidance. The results further provide direct empirical support and added nuance for several risk factors contained in the Stalking Risk Profile.^145^

Again, our findings provide evidence to support the design of preventative programming by detailing the mechanisms via which such attitudes may increase the risk of violent behaviour. We highlight the potential usefulness of assessing individuals’ misogynistic attitudes, alongside their conformity to strict patriarchal gender norms, male entitlement beliefs, hypermasculine attitudes as well as their support for violence against women within existing risk assessment tools. By doing so, we provide program designers with guidance for bespoke risk management plans, on who to target with such programming and how to target different vulnerabilities impacting upon violent (extremist) risk. An aide-memoire or guidance document demonstrating the overlap in misogyny underpinning different types of male violence, and the underlying mechanisms through which misogyny becomes relevant to (extremist) risk, could inform programmatic approaches to preventing violent extremism as well as gender-based violence.

### Limitations and directions for future research

Despite the potential far-reaching implications of the present work, several limitations should be addressed in future research. First, the study is based on data from the U.K., only. As such, the findings may not easily generalise beyond the present study context. Hence, we strongly encourage future studies to examine the associations among misogyny and (extremist) violence in other geographical and socio-cultural/ political contexts. Second, the present sample is only approximately representative based on the following variables: age, gender, and ethnicity. Prolific is an online platform and the subject pool is limited to those who are willing to sign up to the platform. As such, the sample is limited in the extent to which it is truly representative of the general population. We must take this into account when interpreting the findings. Despite these limitations, studies highlight how Prolific affords researchers access to more novel populations than more traditional subject pools like student samples.^146^

Another limitation is the cross-sectional nature of our data. Despite examining multiple theoretically informed moderated mediation models with several violent outcome measures as well as testing for reversed directionality of relationships (i.e., reversed causality), such data does not allow researchers to establish inferences regarding the directionality and causality of the tested direct, indirect, and conditional relationships. It is very likely that many more mechanisms are underlying this relationship and other individual differences are moderating the results, which remain unexplored in our study. The relationship is also likely reciprocal or bi-directional, whereby exposure to violent extremist narratives and settings reinforce personal and group-based grievances against women, which in turn, are fuelling misogynistic attitudes and support for violence against women. Equally, men who hold misogynistic attitudes may be more susceptible to and upon exposure of violent extremism as this may bolster and intensify hypermasculine as well as hostile attitudes towards women. Further studies operationalising longitudinal and experimental research designs are required to establish the temporal direction of the proposed pathways which can speak to cause-and effect-relationships.

Additionally, most correlational statistical models are limited in that they are mainly able to indicate presence, not necessarily relevance. It may be that certain risk factors are highly prevalent in a given population, despite not being relevant to the outcome. Equally, something might occur less frequently in a given population, but in specific instances, it may be highly relevant to the outcome for certain individuals. To explore this, it is key to disentangle between-person from within-person effects, which can only be achieved in carefully designed longitudinal research designs, and ideally to apply person-centred rather than variable-centred statistical approaches. This may eventually result in being able to assess risk for violent extremism and forms of violence against women more comprehensively while further increasing the sensitivity and specificity of risk assessment tools.

We do acknowledge that our analysis is limited as it solely focuses on individual-level risk factors. Yet, to understand how violent (extremist) risk emerges and in order to implement successful intervention strategies for the prevention of VAWG as well as preventing and countering violent extremism, coordinated approaches targeting interactions of multiple risk factors across the social ecology, i.e., on the individual, peer/family, community, and systemic levels, are required.^147^ While beyond the scope of our analysis, approaches that focus upon changing norms on the community and systemic levels provide opportunities to change misogynistic value systems and unhealthy forms of masculinity that promote violence on the individual and interpersonal levels.^148^ By so doing, they attempt to change the mechanisms in society that give rise to and sustain a culture of misogyny and male violence.

There are some shortcomings related to the operationalised measures. For instance, the sample does not consist of people who have engaged, to the best of our knowledge, in gender-based violence or any violent extremist behaviours. Instead, we applied measured participants’ support for gender-based violence and their willingness to engage in violent (extremist) behaviour. Due to ethics approvals and social desirability bias, assessing actual violent (extremist) behaviour is a very challenging to operationalise in general population samples. To mitigate some of these issues, we assessed violent (extremist) intentions (i.e., violent extremist intentions and willingness to engage in interpersonal violence) and endorsement of violence against women rather than actual behaviours. Ajzen’s theory of planned behaviour posits that intentions constitute the immediate antecedents of behaviour and therefore, demonstrate individuals’ readiness to perform a given behaviour.^149^ We further acknowledge that the mean score for the violence against women measure among both genders was unsurprisingly very low in our study. This is likely due to social desirability bias, whereby the actual number of individuals who do support such violence will be higher. In addition, future research would benefit from including a collective narcissism scale and a perceived threat measure which specifically refer to threatened male gender identity.

Lastly, while beyond the scope of the present work, we do acknowledge that women can play a vital part across extremist groups and ideologies by engaging in a multitude of roles and tasks, including involvement in extremist as well as other types of violence. As such, the point we are making is not that women are not involved in violent extremism nor do we argue that they do not commit violence, but rather that the focus here is exclusively on men because the majority of individuals who engage in *violent* extremism and acts of terrorism as well as domestic violence/intimate partner violence, are in fact men. While important qualitative research does point toward the overlap and commonalities underpinning different forms of male violence, rooted in misogyny, patriarchy, and strict masculine gender roles, there is extremely limited quantitative evidence to support this and thus, we consider our study to provide a considerable contribution and extension to existing research. Nevertheless, we do recommend future research to explore the risk factors and underlying mechanisms which drive radicalisation processes among women. Hereby, it will be vital to examine socio-culturally imposed feminine gender roles and identities and their impact on how and why women can equally be susceptible to violent extremist ideologies and why some women also engage in violent extremist behaviours.

## Conclusion

Anecdotal evidence suggests different types of male violence have a shared grounding in extreme patriarchal values, whereby misogyny is complicit in while at the same time also dependent upon violence against women to sustain power structures. Therefore, the main aim of this paper was to provide empirical evidence which speaks to the relevance of misogyny relating to risk towards engagement in different types of violence and to delineate the types of people for whom misogyny may constitute a risk factor for violent extremism and gender-based violence while at the same time identifying some of the mechanisms through which this occurs. Collectively, the study results provide empirical evidence which speaks, to a certain extent, to a common psychology of male violence, driven by a shared hostility and grievances against women, threat perceptions and a sense of victimhood, while at the same time being underpinned by shared mechanisms involving a desire for revenge and hypermasculine norms and behaviours. We provide evidence to suggest that adherence to misogynistic belief systems presents a pertinent risk factor underpinning different types of private and public male violence. This suggests misogyny and its violent manifestations that target women serve as an “ideological” justification that aims to uphold men’s power and dominant status, not just at home but equally within society. Thus, we do not only add to the empirical literature on the interconnectedness of different types of (male) interpersonal violence, but we further provide empirical evidence on the *interconnectedness* of male violence within private and public spheres.^150^

Despite some of the challenges outlined above, more research on the gendered dynamics, i.e., male dominance and female subjugation, as well as gender ideology may offer promising avenues for better understanding the functional role of misogyny and the ways in which the mechanisms link misogyny to violence against women and vulnerability to extremist narratives, groups, and causes. As such, we need to go further and address the underlying social and cultural norms, structures and systems which justify, excuse, or diminish the severity of male-perpetrated aggression and violence, including those which support men’s entitlement to dominance and control over women and those that sustain and reinforce patriarchal gender roles. Importantly, we argue that it is fundamental to apply a gendered framework to better understand the functional role misogyny seems to play in motivating different acts of male violence. Gender-related factors and gendered dynamics seem to play a fundamental role in understanding the mechanisms underpinning different types of male violence.

Relatedly, Phelan calls for a holistic gender analysis framework to enhance our understanding of the different forms of violent extremism and of how gender inequalities impact P/CVE outcomes, and thus, encourages greater integration of gender-sensitive and gender-responsive elements across risk assessment, P/CVE programming design and implementation, as well as policy formulation.^151^ By applying a gender lens, we are likely far better equipped in addressing the impacts of socially imposed gender norms and roles as well as gendered socialisation processes in relation to violence perpetration and gendered radicalisation processes. Recognising the complex and multidimensional nature of gender and the distinct gendered experiences that can shape both men’s and women’s radicalisation processes will be a major step forward.^152^ Continuing to downplay the relationship between gendered dynamics, violence against women and violent extremism and failing to acknowledge the fact that perpetrators of these crimes are predominantly men, will contribute and further sustain a culture of male violence. Therefore, we encourage further research into gender ideology, the gendered dynamics and threat narratives underlying acts of private and public (extremist) violence in order to establish a better understanding of the common psychology fuelling gender-based violence.

## Notes

1. Elizabeth Yardley and Laura Richards, “The Elephant in the Room: Toward an Integrated, Feminist Analysis of Mass Murder,” *Violence Against Women* 29, no. 3–4 (2023): 752–772.

2. Lisa Sugiura, *The Incel Rebellion: The Rise of the Manosphere and the Virtual War against Women* (Emerald Publishing Limited, 2021).

3. For example, see Pablo C. Díaz and Nahla Valji, “Symbiosis of Misogyny and Violent Extremism,” *Journal of International Affairs* 72, no. 2 (2019): 37–56; Joan Smith, *Home Grown: How Domestic Violence Turns Men into Terrorists* (Hachette UK, 2019).

4. Erin E. A. Casey, N. Tatiana Masters, Blair Beadnell, Marilyn J. Hoppe, Diane M. Morrison, and Elizabeth A. Wells, “Predicting Sexual Assault Perpetration among Heterosexually Active Young Men,” *Violence Against Women* 23, no. 1 (2017): 3–27; Christin L. Munsch and Robb Willer, “The Role of Gender Identity Threat in Perceptions of Date Rape and Sexual Coercion,” *Violence Against Women* 18, no. 10 (2012): 1125–1146.

5. Targeted violence refers to acts of violence which are planned and directed at specific individuals, groups, or locations, whereby perpetrators choose their targets to achieve specific motives, such as to redress a grievance or to make a political or ideological statement. See Targeted Violence Prevention Resources for K-12 Schools, *schoolsafety.gov*, April 2023, https://www.schoolsafety.gov/sites/default/files/2023-04/SchoolSafety.gov%20Targeted%20Violence%20Prevention%20Resource%20Infographic_April%202023.pdf (accessed May 20, 2023).

6. Christine Agius, Alexandra Edney-Browne, Lucy Nicholas, and Kay Cook, “Anti-Feminism, Gender and the Far-Right Gap in C/PVE Measures,” *Critical Studies on Terrorism 15*, no. 3 (2022): 681–705; Alexandra Phelan, Melissa Johnston, and Jacqui True, “Growing Threats of Violent Extremism: The Urgency for a Gender-Based Response,” *Australian Institute of International Affairs*, 2020, https://www.internationalaffairs.org.au/australianoutlook/growing-threats-of-violent-extremism-the-urgency-for-a-gender-based-response/ (accessed April 29, 2023); Jessica White, *Gender Mainstreaming in Counter-terrorism Policy: Building Transformative Strategies to Counter Violent Extremism* (Routledge Studies in Gender and Security, 2022).

7. Jude McCulloch, Sandra Walklate, JaneMaree Maher, Kate Fitz-Gibbon, and Jasmine McGowan, “Lone Wolf Terrorism through a Gendered Lens: Men turning Violent or Violent Men Behaving Violently?” *Critical Criminology* 27, (2019): 437–450.

8. Rachel Pain, “Everyday Terrorism: Connecting Domestic Violence and Global Terrorism,” *Progress in Human Geography* 38, no. 4 (2014): 531–550.

9. Also see Caron Gentry, *Disordered Violence: How Gender, Race and Heteronormativity Structure Terrorism* (Edinburgh University Press, 2020).

10. Laura Bates, *Men Who Hate Women: From Incels to Pickup Artists: The Truth About Extreme Misogyny and How It Affects Us All* (Sourcebooks, Inc., 2021); Hadley Freeman, “What do Many Lone Attackers have in Common? Domestic Violence,” *The Guardian*, https://www.theguardian.com/commentisfree/2017/mar/28/lone-attackers-domestic-violence-khalid-masood-westminster-attacks-terrorism (accessed March 29, 2022).

11. McCulloch et al., “Lone Wolf Terrorism through a Gendered Lens.”

12. Ibid., 440; also see Raewyn Connell, “100 Million Kalashnikovs: Gendered Power on a World Scale,” *Debate Feminista* 51, (2016): 3–17; Debbie Ging, “Alphas, Betas, and Incels: Theorizing the Masculinities of the Manosphere,” *Men and Masculinities* 22, no. 4 (2019): 638–657.

13. Caron E. Gentry, “Misogynistic Terrorism: It has always Been Here,” *Critical Studies on Terrorism* 15, no. 1 (2022): 209–224; Elizabeth Pearson, “Gendered Reflections? Extremism in the UK’s Radical Right and al-Muhajiroun Networks,” *Studies in Conflict & Terrorism* 46, no. 4 (2023): 489–512; For a definition of the term “misogynistic incels,” see Megan Kelly, Alex DiBranco, and Julia R. DeCook, “Misogynist Incels and Male Supremacist Violence,” in *Male Supremacism in the United States*, edited by E. K. Carian, A. DiBranco, and C. Ebin (Routledge, 2022): 164–180; Alessia Tranchese and Lisa Sugiura, “‘I Don’t Hate All Women, Just Those Stuck-Up Bitches’: How Incels and Mainstream Pornography Speak the Same Extreme Language of Misogyny,” *Violence Against Women* 27, no. 14 (2021): 2709–2734; Chris Wilson, “Nostalgia, Entitlement and Victimhood: The Synergy of White Genocide and Misogyny,” *Terrorism and Political Violence* 34, no. 8 (2022): 1810–1825.

14. Joshua M. Roose and Joana Cook, “Supreme Men, Subjected Women: Gender Inequality and Violence in Jihadist, Far Right and Male Supremacist Ideologies,” *Studies in Conflict & Terrorism*, advance online publication.

15. Smith, *Home Grown.*

16. Here, we refer to acts of mass violence which were not classified as “terrorism” nor did the perpetrators identify as incels/were not explicitly inspired by incel ideology, but nevertheless, were motivated by a grievance.

17. Everytown For Gun Safety Support Fund, “Mass shootings in the United States: 2009–2016,” 2017, https://everytownresearch.org/wpcontent/uploads/2017/04/Analysis_of_Mass_Shooting_online-pdf-032017.pdf (accessed March 28, 2022).

18. Ruben Vives, Matt Hamilton, and Eric Sondheimer, “After Thousand Oaks Shooting, Picture Emerges of a Troubled Ex-Marine known to Authorities,” *Los Angeles Times*, 2018, https://www.latimes.com/local/lanow/la-me-shooter-profile-20181108-story.html (accessed October 25, 2022).

19. Alexandra Topping, “Maryland Shooting Suspect had Vendetta Against Capital Gazette Paper,” *The Guardian*, 2018, https://www.theguardian.com/us-news/2018/jun/29/maryland-shooting-suspect-jarrod-ramos-capital-gazette (accessed October 25, 2022).

20. Timothy Williams and Amy Harmon, “Maryland Shooting Suspect Had Long-Running Dispute With Newspaper,” *The New York Times*, June 29, 2018, https://www.nytimes.com/2018/06/29/us/jarrod-ramos-annapolis-shooting.html (accessed October 25, 2022).

21. Marganski, “Making a Murderer.”

22. Everytown For Gun Safety Support Fund, “Mass Shootings in the United States: 2009–2016”; Everytown for Gun Safety, “Mass shootings in America 2009–2020,” 2021, https://bit.ly/3fQBlc2 (accessed January 12, 2023); National Threat Assessment Center/United States Secret Service, “Hot Yoga Tallahassee: A Case Study of Misogynistic Extremism,” *Department of Homeland Security* (2022).

23. Kate Manne, “What We get Wrong about Misogyny. Sexism and Misogyny are not the Same—and the Difference Matters” In S. Illing for *Vox*, https://www.vox.com/identities/2017/12/5/16705284/elizabeth-warren-loss-2020-sexism-misogyny-kate-manne (accessed October 22, 2022).

24. Bruce Hoffman, Jacob Ware, and Ezra Shapiro, “Assessing the Threat of Incel Violence,” *Studies in Conflict & Terrorism* 43, no. 7 (2020): 565–587.

25. Lauren Menzie, “Stacys, Beckys, and Chads: The Construction of Femininity and Hegemonic Masculinity within Incel Rhetoric,” *Psychology & Sexuality* 13, no. 1 (2022): 69–85.

26. Maria N. Scaptura and Kaitlin M. Boyle, “Masculinity Threat, ‘Incel’ Traits, and Violent Fantasies among Heterosexual Men in the United States,” *Feminist Criminology* 15, no. 3 (2020): 278–298.

27. Stephane J. Baele, Lewys Brace, and Travis G. Coan. “From ‘Incel’ to ‘Saint’: Analyzing the Violent Worldview behind the 2018 Toronto Attack,” *Terrorism and Political Violence* 33, no. 8 (2021): 1667–1691; Megan Kelly, Alex DiBranco, and Julia R. DeCook, “Misogynist Incels and Male Supremacist Violence,” in *Male Supremacism in the United States*, edited by E. K. Carian, A. DiBranco, and C. Ebin (Routledge, 2022): 164–180; Roberta L. O’Malley, Karen Holt, and Thomas J. Holt, “An Exploration of the Involuntary Celibate (Incel) Subculture Online,” *Journal of interpersonal violence* 37, no. 7–8 (2020): NP4981–NP5008.

28. Brian J. Van Brunt, Bethany S. Van Brunt, Chris Taylor, Nicole Morgan, and Jeffrey Solomon, “The Rise of the Incel Mission-Oriented Attacker,” *Violence and Gender* 8, no. 4 (2021): 163–174.

29. Gentry, *Disordered Violence.*

30. Also see Bates, *Men Who Hate Women.*

31. Simon Cottee, “Incel (E) Motives: Resentment, Shame and Revenge,” *Studies in Conflict & Terrorism* 44, no. 2 (2020): 93–114; Catharina O’Donnell and Eran Shor, “‘This is a Political Movement, Friend’: Why ‘Incels’ ‘Support Violence,’” *The British Journal of Sociolog*y 73, no. 2 (2022): 336–351; Shannon Zimmerman, Lisa Ryan, and David Duriesmith, “Recognizing the Violent Extremist Ideology of ‘Incels,” *Women in International Security Policy Brief*, (2018); Shannon Zimmerman, “The Ideology of Incels: Misogyny and Victimhood as Justification for Political Violence,” *Terrorism and Political Violence*, advance online publication.

32. Clare S. Allely and Lino Faccini, “‘Path to Intended Violence’ Model to Understand Mass Violence in the Case of Elliot Rodger,” *Aggression and Violent Behavior* 37, (2017): 201–209; Kate Manne, *Down Girl: The Logic of Misogyny* (Oxford University Press, 2017); Joshua Thorburn, Anastasia Powell, and Peter Chambers, “A World Alone: Masculinities, Humiliation and Aggrieved Entitlement on an Incel Forum,” *The British Journal of Criminology*, 63, no. 1 (2022): 238–254.

33. Jennifer L. Murray, “The Role of Sexual, Sadistic, and Misogynistic Fantasy in Mass and Serial Killing,” *Deviant Behavior* 38, no. 7 (2017): 735–743; Christopher Vito, Amanda Admire, and Elizabeth Hughes, “Masculinity, Aggrieved Entitlement, and Violence: Considering the Isla Vista mass Shooting,” *Norma* 13, no. 2 (2018): 86–102.

34. Sian Tomkinson, Tauel Harper, and Katie Attwell, “Confronting Incel: Exploring Possible Policy Responses to Misogynistic Violent Extremism,” *Australian Journal of Political Science* 55, no. 2 (2020): 152–169.

35. National Threat Assessment Center/United States Secret Service, “Hot Yoga Tallahassee.”

36. Gentry, *Disordered Violence*; Gentry, “Misogynistic Terrorism.”

37. Gentry, “Misogynistic Terrorism.”

38. Ibid.

39. Gentry, *Disordered Violence;* “Misogynistic Terrorism.”

40. Bates, *Men Who Hate Women*; Manne, *Down Girl*.

41. Gentry, “Misogynistic Terrorism,” 2010.

42. Emily K. Carian, Alex DiBranco, and Chelsea Ebin, eds, *Male Supremacism in the United States: From Patriarchal Traditionalism to Misogynist Incels and the Alt-Right* (Routledge, 2022); Elizabeth Pearson, Emily Winterbotham, and Katherine E. Brown, *Countering Violent Extremism: Making Gender Matter* (Springer Nature, 2021).

43. Hoffman, Ware, and Shapiro, “Assessing the Threat of Incel violence”; Pearson, Winterbotham, and Brown, *Countering Violent Extremism*; Roose and Cook, “Supreme Men, Subjected Women.”

44. Roose and Cook, “Supreme Men, Subjected Women,” 2.

45. Melissa Johnston and Jacqui True, “Misogyny & Violent Extremism: Implications for Preventing Violent Extremism,” *Monash University*, 2019, https://arts.monash.edu/__data/assets/pdf_file/0007/2003389/Policy-Brief_VE_and_VAW_V7t.pdf (accessed January 3, 2023).

46. Díaz and Valji, “Symbiosis of Misogyny and Violent Extremism”; Smith, *Home Grown.*

47. Beth Windisch, “A Downward Spiral: The Role of Hegemonic Masculinity in Lone Actor Terrorism,” *Studies in Conflict & Terrorism*, (2021): 1–18.

48. Greta Jasser, Megan Kelly, and Ann-Kathrin Rothermel, “Male Supremacism and the Hanau Terrorist Attack: Between online Misogyny and Far-right Violence,” *ICCT*, 2021, https://icct.nl/publication/male-supremacism-and-the-hanau-terrorist-attack-between-online-misogyny-and-far-right-violence/ (accessed January 9, 2023).

49. Aage Borchgrevink, *A Norwegian Tragedy: Anders Behring Breivik and the Massacre on Utøya* (Polity, 2013); Wilson, “Nostalgia, Entitlement and Victimhood.”

50. Jason R. Silva, Joel A. Capellan, Margaret A. Schmuhl, and Colleen E. Mills, “Gender-Based Mass Shootings: An Examination of Attacks Motivated by Grievances against Women,” *Violence Against Women* 27, no. 12–13 (2021): 2163–2186.

51. Kayla Allison and Brent R. Klein, “Pursuing Hegemonic Masculinity Through Violence: An Examination of Anti-homeless Bias Homicides,” *Journal of Interpersonal Violence* 36, no. 13–14 (2021): 6859–6882; Mark Follman, Gavin Aronsen, and Deanna Pan, D, “A Guide to Mass Shootings in America,” *Mother Jones*, 2018, https://www.motherjones.com/politics/2012/07/mass-shootings-map/; Marganski, “Making a Murderer,” (accessed January 9, 2023).

52. Silva et al., “Gender-Based Mass Shootings: An Examination of Attacks Motivated by Grievances Against Women,” 2061.

53. Daniel W. Webster, Alexander D. McCourt, Cassandra K. Crifasi, Marisa D. Booty, and Elizabeth A. Stuart, “Evidence Concerning the Regulation of Firearms Design, Sale, and Carrying on Fatal Mass Shootings in the United States,” *Criminology & Public Policy* 19, no. 1 (2020): 171–212; April M. Zeoli and Jennifer K. Paruk, “Potential to Prevent Mass Shootings Through Domestic Violence Firearm Restrictions,” *Criminology & Public Policy* 19, no. 1 (2020): 129–145.

54. Alison J. Marganski, “Making a Murderer: The Importance of Gender and Violence against Women in Mass Murder Events,” *Sociology Compass* 13, no. 9 (2019): e12730.

55. Ellen DeVoe and Jullian Nicholson, “Violence against Women and Public Mass Shooting,” 2020, https://www.bu.edu/ssw/ihsip-blog-violence-against-women-and-public-mass-shootings/ (accessed January 9, 2023).

56. Lisa B. Geller, Marisa Booty, and Cassandr K. Crifasi, “The Role of Domestic Violence in Fatal Mass Shootings in the United States, 2014–2019,” *Injury Epidemiology* 8, no. 38 (2021): 1–8.

57. Everytown for Gun Safety, “Mass Shootings in America 2009–2020.”

58. Díaz and Valji, “Symbiosis of Misogyny and Violent Extremism.”

59. Casey et al., “Predicting Sexual Assault Perpetration Among Heterosexually Active Young Men”; Munsch and Willer, “The Role of Gender Identity Threat in Perceptions of Date Rape and Sexual Coercion”; Robert L. Peralta, Lori A. Tuttle, and Jennifer L. Steele, “At the Intersection of Interpersonal Violence, Masculinity, and Alcohol Use: The Experiences of Heterosexual Male Perpetrators of Intimate Partner Violence,” *Violence Against Women* 16, *no*. 4 (2010): 387–409.

60. McCulloch et al., “Lone Wolf Terrorism Through a Gendered Lens”; Gentry, *Disordered Violence*; Gentry, “Misogynistic Terrorism,” 2010.

61. Agnieszka G. Golec de Zavala, Karolina Dyduch‐Hazar, and Dorottya Lantos, “Collective Narcissism: Political Consequences of Investing Self‐Worth in the Ingroup’s Image,” *Political Psychology 40*, no. S1 (2019): 37.

62. Agnieszka G. Golec de Zavala, Aleksandra Cichocka, Roy Eidelson, and Nuwan Jayawickreme, “Collective Narcissism and its Social Consequences,” *Journal of Personality and Social Psychology* 97, no. 6 (2009): 1074–1096.

63. Golec de Zavala, Dyduch‐Hazar, and Lantos, “Collective Narcissism.”

64. Brian Lickel, Norman Miller, Douglas M. Stenstrom, Thomas F. Denson, and Toni Schmader, “Vicarious Retribution: The Role of Collective Blame in Intergroup Aggression,” *Personality and Social Psychology Review* 10, no. 4 (2010): 372–390.

65. Paulina Górska, Anna Stefaniak, Katarzyna Malinowska, Katarzyna Lipowska, Marta Marchlewska, Magdalena Budziszewska, and Oliwia Maciantowicz, “Too Great to Act in Solidarity: The Negative Relationship Between Collective Narcissism and Solidarity-based Collective Action,” *European Journal of Social Psychology* 50, no. 3 (2019): 561–578; Agnieszka G. Golec de Zavala and Kinga Bierwiaczonek, “Male, National, and Religious Collective Narcissism Predict Sexism,” *Sex Roles* 84, (2020): 680–700.

66. Katarzyna Jásko, David Webber, Arie W. Kruglanski, Michele Gelfand, Muh Taufiqurrohman, Malkanthi Hettiarachchi, and Rohan Gunaratna, “Social Context Moderates the Effects of Quest for Significance on Violent Extremism,” *Journal of Personality and Social Psychology* 118, no. 6 (2020): 1165–1187.

67. Magdalena A. Źemojtel-Piotrowska, … Eduardo Wills-Herrera, “Measurement of Psychological Entitlement in 28 Countries,” *European Journal of Psychological Assessment 33*, no. 3 (2017): 207–217.

68. Clark McCauley and Sophia Moskalenko, “Mechanisms of Political Radicalization: Pathways Toward Terrorism,” *Terrorism and Political Violence* 20, no. 3 (2008): 415–433.

69. Dorothy Ayers Counts, “Female Suicide and Wife Abuse: A Cross‐Cultural Perspective,” *Suicide and Life‐Threatening Behavior* 17, no. 3 (1987): 194–204.

70. Diana Scully and Joseph Marolla, “‘Riding the Bull at Gilley’s: Convicted Rapists Describe the Rewards of Rape,” *Social Problems* 32, no. 3 (1985): 251–263.

71. Karolina Dyduch-Hazar and Blazej Mrozinski, “Opposite Associations of Collective Narcissism and In-group Satisfaction with Intergroup Aggression via Belief in the Hedonistic Function of Revenge,” *Plos one* 16, no. 3 (2021): e0247814; Rahav Gabay, Boaz Hameiri, Tammy Rubel-Lifschitz, and Arie Nadler, “The Tendency for Interpersonal Victimhood: The Personality Construct and its Consequences,” *Personality and Individual Differences* 165, (2020): 110,134.

72. James A. Fox, Jack Levin, and Emma E. Fridel, E. E., *Extreme Killing: Understanding Serial and Mass Murder* (SAGE, 2018).

73. Murray, “The Role of Sexual, Sadistic, and Misogynistic Fantasy in Mass and Serial Killing.”

74. Alannah J. Cooper, Michele T. Pathé, and Troy E. McEwan, “The Role of Grievance in Fatal Family Violence and Implications for the Construct of Lone Actor Grievance-Fuelled Violence,” *Frontiers in Psychology* 13, (2022): 1,057,719.

75. Rachel Kalish and Michael Kimmel, “Suicide by Mass Murder: Masculinity, Aggrieved Entitlement, and Rampage School Shootings,” *Health Sociology Review* 19, no. 4 (2010): 451–464.

76. Marganski, “Making a Murderer.”

77. Richard M. Eisler, Joseph J. Franchina, Todd M. Moore, Hunter G. Honeycutt, and Deborah L. Rhatigan, “Masculine Gender Role Stress and Intimate Abuse: Effects of Gender Relevance of Conflict Situations on Men’s Attributions and Affective Responses,” *Psychology of Men & Masculinity* 1, no. 1 (2000): 30–36; Munsch and Willer, “The Role of Gender Identity Threat in Perceptions of Date Rape and Sexual Coercion.”

78. Donald L. Mosher and Markin Sirkin, “Measuring a Macho Personality Constellation,” *Journal of Research in Personality* 18, no. 2 (1984): 150–163.

79. Dennis E. Reidy, Danielle S. Berke, Brittany Gentile, and Amos Zeichner, “Man Enough? Masculine Discrepancy Stress and Intimate Partner Violence,” *Personality and Individual Differences* 68, (2014): 160–164; Robb Willer, Christabel L. Rogalin, Bridget Conlonand Michael T. Wojnowicz, “Overdoing Gender: A Test of the Masculine Overcompensation Thesis,” *American Journal of Sociology* 118, *no*. 4 (2013): 980–1022.

80. Joseph A. Vandello, Jennifer K. Bosson, Dov Cohen, Rochelle M. Burnaford, and Jonathan R. Weaver, “Precarious Manhood,” *Journal of Personality and Social Psychology* 95, no. 6 (2008): 1325–1339.

81. Jennifer K. Bosson, Joseph A. Vandello, Rochelle M. Burnaford, Jonathan A. Weaver, and S. Arzu Wasti, “Precarious Manhood and Displays of Physical Aggression,” *Personality and Social Psychology Bulletin* 35, no. 5 (2009): 623–634.

82. Anne Maass, Mara Cadinu, Gaia Guarnieri, and Annalisa Grasselli, “Sexual Harassment under Social Identity Threat: The Computer Harassment Paradigm,” *Journal of Personality and Social Psychology* 85, no. 5 (2003): 853–870.

83. Allison and Klein, “Pursuing Hegemonic Masculinity through Violence”; Messerschmidt, “Masculinities and Femicide.”

84. Maria N. Scaptura, Brittany E. Hayes, Jeffrey Gruenewald, William S. Parkin, and Michelle E. Protas, “The Perfect Aryan Housewife”: Intersection of Extremist Violence and Violence Against Women,” *Crime & Delinquency* 69, no. 3 (2023): 607–629.

85. Ibid.

86. It is important to note that our data is cross-sectional and therefore we cannot draw causal inferences. The ordering of the determinants in our proposed models is guided by previous research. Therefore, neither the direction of the individual hypotheses nor the exact ordering of the constructs can be determined with our data, instead they are theoretically informed to test the proposed models.

87. See note 3 above.

88. Yves Rosseel, *The Lavaan Tutorial* (Belgium: Ghent University), https://lavaan.ugent.be/tutorial/ (accessed June 25, 2023).

89. Kristopher J. Preacher, Derek D. Rucker, and Andrew F. Hayes, “Addressing Moderated Mediation Hypotheses: Theory, Methods, and Prescriptions,” *Multivariate Behavioral Research* 42, no. 1 (2007): 185–227.

90. Andrew F. Hayes, *Introduction to Mediation, Moderation, and Conditional Process Analysis: A Regression-Based Approach* (Guilford Publications, 2017).

91. Ibid.

92. Ibid.

93. Bettina Rottweiler and Paul Gill, “‘Measuring Individuals’ Misogynistic Attitudes: Development and Validation of the Misogyny Scale,” *PsyArXiv* (Preprint), https://psyarxiv.com/6f829.

94. Thomas F. Denson, William C. Pedersen, and Norman Miller, “The Displaced Aggression Questionnaire,” *Journal of Personality and Social Psychology* 90, no. 6 (2006): 1032–1051.

95. Julian S. Walker, “The Maudsley Violence Questionnaire: Initial Validation and Reliability,” *Personality and Individual Differences* 38, no. 1 (2005): 187–201.

96. Mosher and Sirkin, “Measuring a Macho Personality Constellation.”

97. Golec de Zavala et al., “Collective Narcissism and its Social Consequences.”

98. Golec de Zavala, Dyduch‐Hazar, and Lantos, “Collective Narcissism.”

99. Golec de Zavala et al., “Collective Narcissism and its Social Consequences.”

100. Sophia Moskalenko and Clark McCauley, “Measuring Political Mobilization: The Distinction between Activism and Radicalism,” *Terrorism and Political Violence* 21, no. 2 (2009): 239–260.

101. Bettina Rottweiler, Paul Gill, and Noemie Bouhana, “Individual and Environmental Explanations for Violent Extremist Intentions: A German Nationally Representative Survey Study,” *Justice Quarterly* 39, no. 4 (2022): 825–846.

102. Walker, “The Maudsley Violence Questionnaire.”

103. For example see Marganski, “Making a Murderer.”

104. For example see Roose and Cook, “Supreme Men, Subjected Women.”

105. For example see Debbie Ging, “Alphas, Betas, and Incels: Theorizing the Masculinities of the Manosphere,” *Men and Masculinities* 22, no. 4 (2019): 638–657.

106. Golec de Zavala and Bierwiaczonek, “Male, National, and Religious Collective Narcissism Predict Sexism.”

107. Ibid.

108. Golec de Zavala et al., “Collective Narcissism and its Social Consequences.”

109. Dyduch-Hazar and Mrozinski, “Opposite Associations of Collective Narcissism and In-group Satisfaction with Intergroup Aggression via Belief in the Hedonistic Function of Revenge.”

110. Jásko et al., “Social Context Moderates the Effects of Quest for Significance on Violent Extremism.”

111. Center on Extremism, “When Women are the Enemy: The Intersection of Misogyny and White Supremacy,” *ADL Center on Extremism Report*, 2018; Alex DiBranco, “Male Supremacist Terrorism as a Rising Threat,” *International Centre for Counter-Terrorism–The Hague* 10, (2010), https://icct.nl/publication/male-supremacist-terrorism-as-a-rising-threat/.

112. Díaz and Valji, “Symbiosis of Misogyny and Violent Extremism”; Michael S. Kimmel, *Angry White Men: American Masculinity at the End of an Era* (Hachette UK, 2017); Michael S. Kimmel, *Healing from Hate: How Young Men Get Into—and Out Of—Violent Extremism* (Berkeley: University of California Press, 2018).

113. Joshua M. Roose, “‘Ideological Masculinity’ that Drives Violence Against Women is a Form of Violent Extremism,” *The Conversation*, (2018), https://theconversation.com/ideological-masculinity-that-drives-violence-against-women-is-a-form-of-violent-extremism-99603.

114. Allison and Klein, “Pursuing Hegemonic Masculinity through Violence”; James W. Messerschmidt, “The Salience of ‘Hegemonic Masculinity,’” *Men and Masculinities* 22, no. 1 (2019): 85–91; Bosson et al., “Precarious Manhood and Displays of Physical Aggression”; Vandello et al., “Precarious Manhood.”

115. Joshua M. Roose, Michael Flood, Alan Greig, Mark Alfano, and Simon Copland, *Masculinity and Violent Extremism* (Palgrave MacMillan, 2022).

116. Gentry, “Misogynistic Terrorism”; Brynjar Lia, “The Jihādī Movement and Rebel Governance: A Reassertion of a Patriarchal Order?” *Die Welt des Islams* 57, no. 3–4 (2017): 458–479.

117. Roose et al., *Masculinity and Violent Extremism.*

118. Ruth Lewis, Mike Rowe, and Clare Wiper, “Online/Offline Continuities: Exploring Misogyny and Hate in Online Abuse of Feminists,” in *Online Othering: Exploring Digital Violence and Discrimination on the Web*, edited by K. Lumsden and Emily Harmer (Springer, 2022): 121–143; Karen Lumsden and Heather Morgan, “Media Framing of Trolling and Online Abuse: Silencing Strategies, Symbolic Violence, and Victim Blaming,” *Feminist Media Studies* 17, no. 6 (2017): 926–940; Lisa Sugiura and April Smith, “Victim Blaming, Responsibilization and Resilience in Online Sexual Abuse and Harassment,” in *Victimology: Research, Policy and Activism*, edited by J. Tapley and P. Davies (Palgrave Macmillan, 2020): 45–79.

119. Sherry Hamby and John Grych, *The Web of Violence: Exploring Connections Among Different Forms of Interpersonal Violence and Abuse* (Springer Science & Business Media, 2012).

120. Hamby and Grych, “The Web of Violence; Senkans, Relationship Cognition in Intimate Partner Violence and Post-Relationship Stalking” (Doctoral Thesis, 2017).

121. Svenja Senkans, Troy E. McEwan, T. E., and James Ogloff, “Conceptualising Intimate Partner Violence Perpetrators’ Cognition as Aggressive Relational Schemas,” *Aggression and Violent Behavior* 55, (2020): 101,456.

122. Paul Gill, *Lone-actor Terrorists: A Behavioural Analysis* (Routledge, 2015).

123. Michael Flood and Bob Pease, “Factors Influencing Attitudes to Violence against Women,” *Trauma, Violence, & Abuse* 10, no. 2 (2009): 125–142; Flood and Pease’s study shows a robust effect of men’s adherence to sexist, patriarchal, and/or sexually hostile attitudes on the use of violence against women.

124. Violence Prevention Network and the Centre for Feminist Foreign Policy, “How Anti-Feminist and Anti-Gender Ideologies Contribute to Violent Extremism—and What We can do About It,” *Centre for Feminist Foreign Policy*, 2021, https://static1.squarespace.com/static/57cd7cd9d482e9784e4ccc34/t/61eec9ad3ad0a2796c147d07/1643039149698/CFFP2-1.pdf.

125. Gentry, “Misogynistic Terrorism”; Alexandra Phelan, “Special Issue Introduction for Terrorism, Gender and Women: Toward an Integrated Research Agenda,” *Studies in Conflict and Terrorism* 46, no. 4 92,023): 353–361; Roose and Cook, “Supreme Men, Subjected Women.”

126. Home Office, *Counter‐terrorism Strategy (CONTEST)* (London, 2018).

127. Department of Homeland Security, “Center for Prevention Programs and Partnerships” 2021, https://www.dhs.gov/CP3.

128. Gender transformation is defined “[…] as educating for gender respect and justice through fostering boys’ and men’s critical understandings about gender inequality, masculinity and gender-based violence.” See Amanda Keddie, Michael Flood, and Shelley Hewson-Munro, “Intersectionality and Social Justice in Programs for Boys and Men,” *NORMA* 17, no. 3 (2023): 2; also see Our Watch, *Men in Focus: Unpacking Masculinities and Engaging men in the Prevention of Violence Against Women* (Melbourne, 2019); Michael Flood, *Engaging Men and Boys in Violence Prevention* (Basingstoke: Palgrave Macmillan).

129. Flood, *Engaging Men and Boys in Violence Prevention*; Keddie, Flood, and Hewson-Munro, “Intersectionality and Social Justice in Programs for Boys and Men.”

130. Melissa Johnston and Sara Meger, “Linkages between Violent Misogyny and Violent Extremism and Radicalization that Lead to Terrorism,” *The Organisation for Security and Cooperation in Europe*, (2022); Pearson, Winterbotham, and Brown, *Countering Violent Extremism*.

131. Rachel Jewkes, Michael Flood, M., and James Lang, “From Work With Men and Boys to Changes of Social Norms and Reduction of Inequities in Gender Relations: A Conceptual Shift in Prevention of Violence Against Women and Girls,” *The Lancet* 385, no. 9977 (2015): 1580–1589.

132. Caitlin Clemmow, Bettina Rottweiler, and Paul Gill, “Rethinking the Dichotomy Between Domestic Abuse and Violent Extremism,” (under review).

133. Keddie, Flood, and Hewson-Munro, “Intersectionality and Social Justice in programs for Boys and Men”; Our Watch, *Men in Focus*.

134. Bettina Rottweiler & Paul Gill, “The Formation of Grievances and their Impact upon Violent Extremist Intentions,” (under review).

135. Bettina Rottweiler and Paul Gill, “Individual Differences in Personality Moderate the Effects of Perceived Group Deprivation on Violent Extremism: Evidence from a United Kingdom Nationally Representative Survey,” *Frontiers in Psychology* 13, no. 99 (2023).

136. Bettina Rottweiler, *Risk- and Protective Factors for Violent Extremist Intentions* (Doctoral dissertation, UCL, 2022); Bettina Rottweiler and Paul Gill, “Conspiracy Beliefs and Violent Extremist Intentions: The Contingent Effects of Self-Efficacy, Self-Control and Law-Related Morality,” *Terrorism and Political Violence* 34, no. 7 (2022), 1485–1504.

137. The Home Office, “Channel: Vulnerability Assessment Framework,” https://www.gov.uk/government/publications/channel-vulnerability-assessment.

138. Ellen Pressman, Nils Duits, Thomas Rinne, and John Flockton, “VERA-2R Violent Extremism Risk Assessment Version 2 Revised: A Structured Professional Judgment Approach,” (Utrecht, The Netherlands: NIFP/DJI, 2016.

139. For the structural properties see Beverly Powis, Kiran Randhawa, and Darren Bishopp, “An Examination of the Structural Properties of the Extremism Risk Guidelines (ERG22+): A Structured Formulation Tool for Extremist Offenders,” *Terrorism and Political* Violence 33, no. 6 (2019): 1141–1159.

140. Monica Lloyd, “Extremist Risk Assessments: A Directory,” *CREST*, 2019, https://crestresearch.ac.uk/resources/extremism-risk-assessment-directory/ (accessed January 15, 2023).

141. Jodi L. Viljoen, Keith R. Cruise, Tonia L. Nicholls, Sarah L. Desmarais, and Christopher D. Webster, “Taking Stock and Taking Steps: The Case for an Adolescent Version of the Short-Term Assessment of Risk and Treatability,” *International Journal of Forensic Mental Health* 11, no. 3 (2012): 135–149.

142. Randy Borum, Henny P. Lodewijks, Patrick A. Bartel, and Adelle E. Forth, “The Structured Assessment of Violence Risk in Youth (SAVRY),” in *Handbook of Violence Risk Assessment*, edited by K. S. Douglas and R.K. Otto, (Routledge, 2020): 438–461; Bosson et al., “Precarious Manhood and Displays of Physical Aggression.”

143. P. Randall Kropp and Andrea Gibas, “The Spousal Assault Risk Assessment Guide (SARA),” in *Handbook of Violence Risk Assessment*, edited by K. S. Douglas and R.K. Otto, (Routledge, 2020): 389–409.

144. Kevin S. Douglas, Stephen D. Hart, Christopher D. Webster, and Henrik Belfrage, *HCR-20V3: Assessing Risk for Violence: User Guide* (Mental Health, Law, and Policy Institute, Simon Fraser University, 2013).

145. Rachel MacKenzie, Troy E. McEwan, Michele Pathe, David V. James, James R. Ogloff, and Paul E. Mullen, *Stalking Risk Profile: Guidelines for the Assessment and Management of Stalkers* (Monash University, 2009).

146. Eyal Peer, Laura Brandimarte, Sonam Samat, and Alessandro Acquisti, “Beyond the Turk: Alternative Platforms for Crowdsourcing Behavioral Research,” *Journal of Experimental Social Psychology* 70, (2017): 153–163.

147. Noemie Bouhana, *The Moral Ecology of Extremism: A Systemic Perspective* (Commission for Countering Extremism, 2019); Jewkes, Flood, and Lang, “From Work with Men and Boys to changes of Social Norms and Reduction of Inequities in Gender Relations.”

148. Keddie, Flood, and Hewson-Munro, “Intersectionality and Social Justice in Programs for Boys and Men”; Our Watch, *Men in Focus*.

149. Icek Ajzen, “From Intentions to Actions: A Theory of Planned Behaviour,” in *Action Control: From Cognition to Behaviour*, edited by J. Kuhl and J. Beckmann (Berlin Heidelberg: Springer): 1–39.

150. John Gryc and Sherry Swan, “Toward a More Comprehensive Understanding of Interpersonal Violence: Introduction to the Special Issue on Interconnections Among Different Types of Violence,” *Psychology of Violence 2*, no. 2 (2012): 105–110; Hamby and Grych, *The Web of Violence.*

151. Phelan, “Special Issue Introduction for Terrorism, Gender and Women.”

152. Alexandra Phelan, James Paterson, Jessica White, and Claudia Wallner, “Misogyny, Hostile Beliefs and the Transmission of Extremism: A Comparison of the Far-Right in the UK and Australia” (CREST, 2023), https://crestresearch.ac.uk/comment/misogyny-and-masculinity-toward-a-typology-of-gendered-narratives-amongst-the-far-right/ (accessed September 10, 2023).

